# Levels of diphtheria and tetanus specific IgG of Portuguese adult women, before and after vaccination with adult type Td. Duration of immunity following vaccination

**DOI:** 10.1186/1471-2458-7-109

**Published:** 2007-06-12

**Authors:** Guilherme Gonçalves, Maria Augusta Santos, João Graça Frade, José Saraiva Cunha

**Affiliations:** 1 Instituto Gulbenkian de Ciência, Oeiras, Portugal; 2 Instituto de Ciências Biomédicas de Abel Salazar, Universidade do Porto, Portugal; 3 Instituto Nacional de Saúde Dr. Ricardo Jorge, Delegação no Porto, Portugal; 4 Centro de Saúde de Condeixa-a-Nova, Coimbra, Portugal; 5 Faculdade de Medicina, Universidade de Coimbra, Portugal

## Abstract

**Background:**

The need for tetanus toxoid decennial booster doses has been questioned by some experts. Several counter arguments have been presented, supporting the maintenance of decennial adult booster doses with tetanus and diphtheria toxoids (adult formulation of the vaccine: Td). This study aimed to evaluate the use of Td in Portuguese adult women under routine conditions. For that purpose we selected a group of women 30+ years of age to which vaccination was recommended. We intended to know if pre-vaccination antibody concentrations were associated with factors as age at first and last vaccination, number of doses and time since last revaccination. We also intended to assess the serological efficacy of Td booster.

**Methods:**

Following the Portuguese guidelines 100 women were vaccinated with Td. Antitetanus toxin IgG (ATT IgG) and antidiphtheria toxin IgG (ADT IgG) levels were measured (mIU/ml) in 100 pre-vaccination and 91 post-vaccination sera. Detailed vaccination records were available from 88 participants.

**Results:**

Twenty-two women (Group A) began vaccination with DPT/DT in their early childhood and their pre-vaccination ATT IgG levels increased with the number of doses received (p = 0.022) and decreased with time since last vaccination (p = 0.016). Among the 66 women who began vaccination in adolescence and adulthood (Group B), with monovalent TT, ATT IgG levels decreased with age at first dose (p < 0.001) and with time since last vaccination (p = 0.041). In Group A, antidiphtheria toxin IgG kinetics was very similar to that observed for ATT IgG. Among women not vaccinated with diphtheria toxoid, ADT IgG levels decreased with age. Serological response to both components of Td was good but more pronounced for ATT IgG.

**Conclusion:**

Our study suggests that, to protect against tetanus, there is no need to administer decennial boosters to the Portuguese adults who have complied with the childhood/adolescent schedule (6 doses of tetanus toxoid). The adult booster intervals could be wider, probably of 20 years. This also seems to apply to protection against diphtheria, but issues on the herd immunity and on the circulation of toxigenic strains need to be better understood.

## Background

Tetanus and diphtheria toxoids have been used in different vaccine formulations, to immunise against tetanus and diphtheria [1,2]. DPT and DT (diphtheria-tetanus-pertussis and diphtheria-tetanus vaccines) have been used in primary immunisation in early childhood [1,2].

The degree and duration of immunity against tetanus increases with the number of doses of tetanus toxoid given [3] and there is a continuous decline in antitetanus toxin serum concentration after primary vaccination as well as revaccination [4]. Three doses of tetanus toxoid are necessary for primary immunisation, in early childhood as later in life (adults) [3,4] and many countries have recommend the use of 3 doses of DPT in early childhood [5,6]. Revaccination strategies and schedules vary widely [7].

For diphtheria, it was more difficult to draw conclusions about the effect of primary vaccination, because response was influenced by naturally acquired immunity [4]. Studies in populations with little or no diphtheria have shown that antibody kinetics after vaccination is similar to that observed for tetanus vaccination [4].

For many years, several countries have recommended decennial boosters to adults, using the monovalent tetanus vaccine (TT) [5,6].

Initial attempts to use booster doses of diphtheria toxoid were associated with high rates of adverse reactions [2,8] and vaccination programmes did not include adult boosters [5]. To overcome that problem, a Td combination (with a lower amount of diphtheria toxoid) was eventually developed for use in adults [9]. Meanwhile, diphtheria epidemics occurred in the former Soviet Union states [2,10,11]. Coupled with serological data showing low levels of protection among adults in several West European countries [2,11,12] that epidemic raised the concerns of resurgence of diphtheria in those countries, leading to the recommendation to replace TT by Td in the adult decennial boosters [2,11].

The need for tetanus toxoid decennial booster doses has been questioned by some experts [1,4,9,13-15]. The main argument was that few cases of tetanus have been observed among people who received the primary vaccination series, regardless they had received booster doses [1]. Further arguments against decennial boosters include cost-effectiveness considerations [14] and the concerns with post Td adverse reactions [9]. As an alternative it has been proposed in the USA that, following a primary childhood series and teenage/young adult booster, no further boosters should be given until 50 years of age, except as part of procedures recommended in wound management [1,9]. This lead to an interesting situation in the USA, where the Advisory Committee on Immunization Practices has continued to recommend boosters every 10 years, but in the official guidelines [15] it is also mentioned the alternative proposed by the American College of Physicians.

Central to this discussion have been the studies of Simonsen, in Denmark [4,16]. He measured antitetanus toxin IgG (ATT IgG) serum levels and used linear regression models to assess the duration of immunity after vaccination with primary series and after revaccination [4,16]. Consequent to his observations, Simonsen proposed to the Danish population a school age booster after primary immunization, followed by routine boosters every 20 years [1,16].

Several counter arguments have been presented, supporting the maintenance of decennial adult booster doses with Td [1]. It was argued that the recommendations to the Danish population could lead to significant proportions of unprotected adults in other populations [1]. The rationale for decennial tetanus toxoid boosters is to maintain serum ATT IgG well above protective levels for nearly all members of the population [1]. Furthermore, severe cases and deaths due to tetanus were observed among previously vaccinated people, and older adults may not have as great or long lasting responses to a single booster as younger individuals [1,17]. Post-vaccination antidiphtheria toxin IgG (ADT IgG) levels decline more rapidly than ATT IgG levels, which has been considered "the final consideration" to support decennial boosters with Td [1].

In Portugal, although vaccination had been available for years, a comprehensive, nation-wide vaccination program (NVP) only began in 1966 [18]. Primary vaccination with 3 doses of DPT was recommended in the first year of life [18,19]. Booster doses of DPT were recommended at age 18–24 months and 5–6 years [19,20]. Booster doses with TT were recommended at 11–13 years of age and every 10 years afterwards [20]. DT vaccine was recommended as an alternative to DPT for those reporting serious adverse reactions after DPT or for primary vaccination of children aged 7–9, while TT should be given to adults to complete the primary series [19]. For adults without vaccination records or who had never received tetanus toxoid, primary vaccination was recommended, with 3 doses, followed by a booster 6–12 months after the third dose; for women in childbearing age, an additional booster (5^th ^dose) was recommended 1–5 years after the third dose; after that, decennial boosters were recommended for both sexes [20].

Since 2001, Td has replaced the TT in adolescent and adult decennial boosters, and in primary vaccination of adults who need it [20]. This change was influenced by the evidence published in the international literature and by a study among Portuguese recruits [12].

In January 2006, DPwT (with whole cell pertussis component) was replaced by a DPaT (acellular pertussis); the number of doses and recommended schedule was kept; Td has also replaced DT, for primary immunisation for those 7+ years [21].

We have only studied women because men are vaccinated when they are conscripts [12] and those booster doses are seldom recorded in vaccination records.

This study aimed to evaluate the use of Td in Portuguese adult women under routine conditions. For that purpose we selected a group of women 30+ years of age to which Td vaccination was recommended by the NVP guidelines. We intended to know if pre-vaccination antibody concentrations were associated with factors as age at first and last vaccination, number of doses and time since last revaccination. We also intended to assess the serological efficacy of Td booster.

## Methods

### Study design and enrolment

From the computerised records of two General Practitioners, we identified 324 women 30+ years of age, who had received tetanus vaccine more than 10 years before or who had no vaccinations recorded. The computerised database included information on vaccination, transcribed from the written individual vaccination records kept in the vaccination room of the health centre (HC) of Condeixa-a-Nova, Coimbra, Portugal. All operational and ethical aspects of the study were authorized by the Portuguese National Health Service coordinating board, at district level (*Sub-Região de Saúde de Coimbra*), after a detailed written study protocol had been submitted.

Those women were contacted by telephone and asked to attend the HC to be vaccinated with Td. They were asked to bring their individual vaccination booklet. If a telephone number was not available or they did not answer the calls, they were sent a post card. When attending the vaccination room, their vaccination booklet was checked against the written record in the HC. Participants signed an informed consent, had a blood sample collected by venupuncture and were vaccinated: the 100 participants were given an intradeltoid injection of 0.5 ml of *ANATOXAL Di Te Berna Adultos*^® ^(batch 8500005) containing purified tetanus-toxoid (corresponding to a potency ≥20 IU) and diphtheria-toxoid (potency ≥2 IU), adsorbed to aluminum hydroxide; in the remaining text we just mention that women were vaccinated with "Td".

The first 109 women attending the vaccination room agreed to participate but nine were excluded because they presented updated vaccination records showing they did not need to be revaccinated. Women without individual vaccination booklets were stimulated to bring them to the HC, still after being vaccinated with Td, should they find those records. One hundred and ten women never attended the HC to be vaccinated. After the intended number of 100 vaccinated participants was recruited, women attending the vaccination room just received Td as recommended [20] and we did not collect data about them.

### Laboratory procedures

Blood samples were allowed to clot at room temperature and then serum was separated, frozen, and transported in appropriate containers to the lab where they were kept at -70°C till testing. All samples were studied at the Serology Laboratory of the National Institute of Health, in Porto, Portugal. Antitetanus toxin IgG (ATT IgG) was measured using a commercial enzyme immunoassay (EIA) (*Tetanus ELISA IgG Testkit*^®^, *Genzyme Virotech GmbH*). Using a calibration curve, optical density (OD) measurements were automatically converted into mIU/ml. Each serum was tested twice and the result was the arithmetic mean of the two measurements (in mIU/ml). Following the recommendations of the EIA manufacturer, sera were also given a qualitative classification. For Antidiphtheria toxin IgG (ADT IgG) the lab procedures were identical but using the commercial EIA (*Diphtheria Tetanus ELISA IgG Testkit*^®^, *Genzyme Virotech GmbH*).

ATT IgG levels ≥160 mIU/ml were considered protective, because such threshold has been recommended [3] and used [22] when EIA technique is performed; according to that threshold, women were classified as "immune" or "susceptible" to tetanus.

ADT IgG levels ≥100 mIU/ml were considered protective, because such threshold has been recommended [23] and used [24] when EIA technique is performed; according to that threshold, women were classified as "immune" or "susceptible" to diphtheria. Additionally, we considered those with levels ≥1000 mIU/ml has having "long-term protection" [2,25].

### Data analysis and statistical methods

Data were entered twice by independent operators and validated using Epi Info 6.04 d [26], and analysed using Stata [27] software.

The total number of tetanus toxoid and diphtheria toxoid doses received by each woman was used as an independent variable in different analysis. Moreover we considered the variable "primary vaccination" or "primary series" (yes/no) for both tetanus and diphtheria vaccination depending on having received 3+ doses of the corresponding toxoid in their lifetime.

Antibody concentration distributions in pre and post-vaccination sera, and the corresponding concentration differences were log-transformed to obtain a more closely normal distribution to be used in the statistical analysis. Thus, geometric mean concentration (GMC) values reported in the text in mIU/ml correspond to anti-logs. Few pre and post-vaccination sera had no detectable levels of ADT IgG (concentration = 0 mIU/ml); to enable the log-transformation, their inclusion in the regression models and the graphic display, we allocated those sera the fake concentration value of 1 mIU/ml of ADT IgG. Simple linear and multiple regression models [28] were used to assess associations between antibody concentration levels and differences between post and pre-vaccination levels, as dependent variables, and number of vaccine doses, time since last vaccination and age at first (and at last) vaccination, as independent variables. Analysis of variance (Anova) was used to compare GMC between groups. Qui-squared (χ^2^) test was used to compare proportions between groups.

## Results

Participant's mean age was 57.8 years (range 29.8–97.5 years, S.D. = 17.9). We do not have data on the reproductive life of women. Socioeconomic or clinical data were not known. Initial blood sample collection (n = 100) and vaccination, took place between May 15 and July 3, 2003. Post-vaccination blood samples were collected from 91 women, in the HC, 46 to 96 days after vaccination (mean = 64 and S.D. = 11 days).

### Vaccination status

Td vaccine was given to the 100 participants, as recommended by the Portuguese guidelines [20]: eighty-one participants had received the last dose of tetanus-toxoid more than 10 years before; initially, vaccination history was unknown for 19 women. Some weeks latter, 7 of these women presented written vaccination records showing that they had received tetanus toxoid less than 10 years before; 5 of those had received less than 4 doses in their life. After this update, we ended up with 12 women without known vaccination history and 88 participants for whom we knew types and numbers of vaccines received at which exact dates. Among those without known vaccination history, one was 66 and the others 77+ years of age.

The analysis of the vaccination history revealed two clear patterns: some women were vaccinated in early childhood with DPT (or DT) while others only began vaccination later in life (with TT).

Twenty two women (Group A; n = 22) had begun their vaccination against tetanus in childhood, receiving either DPT before the age of 7 (n = 20) or DT, between 7–9 years of age (n = 2); women in Group B (n = 66) began their vaccination with TT after 10 years of age but only 4 were vaccinated before their 20^th ^birthday. Table 1 shows the distribution of the two groups by age (and years of birth) and vaccination history. Women in Group A were less than 50 years of age and they were born between 1956 and 1973 while women in Group B were significantly older (Table 1) and were born between 1913 and 1971. In Group B, 85% of women were 10+ years of age when the NVP began while in the younger Group A, none of the 9 women born before 1966 was 10 by the time the NVP began (in 1966). Nevertheless, in 7 women of Group A, the first dose of tetanus and diphtheria toxin was given before 1966, while that was only recorded in one women from Group B. Only 59.1% (39/66) of women in Group B completed primary vaccination (3+ doses). All women in Group A completed primary vaccination against tetanus and diphtheria. All 20 women who had begun vaccination with DPT completed the primary series with the same vaccine, 11 in the first year of life and 9 before 7 years of age. The 2 women, who began vaccination with DT, completed the primary series when they were 11 and 18 years of age. By contrast, Group B began vaccination in a much older age (Table 1), and among the 39 women who completed primary vaccination against tetanus, 37 did it after their 30^th ^birthday. Women who began vaccination with DPT/DT received more lifetime doses of tetanus toxoid (Table 1); 2 of those had received up to 9 doses.

**Table 1:** Distribution of women who began vaccination with DPT/DT or TT, by age and vaccination history

Variable	Value	Group A: Began vaccination with DPT/DT (n = 22)	Group B: Began vaccination with TT (n = 66)	p value
Years of birth				-
	1910 – 1944	0	30	
	1945 – 1954	0	24	
	1955 – 1964	8	9	
	1965 – 1974	14	3	
Age group (in years)				<0.0001
	30 – 39	14	3	
	40 – 49	8	13	
	50 – 59	0	25	
	60 – 69	0	12	
	70 – 99	0	13	
N.° doses of tetanus toxoid				<0.0001
	1	0	15	
	2	0	12	
	3	0	31	
	4	2	2	
	5	3	6	
	6–9	17	0	
Age at first dose				<0.0001
	Mean	2.2	41.2	
	S.D.	2.6	15.7	
	Min/Max	0.3/7.9	11.1/77.0	
Time since last dose (years)				0.86
	Mean	15.8	14.4	
	S.D.	7.3	6.0	
	Min/Max	5.0/34.4	1.6/33.0	

Only women from group A were vaccinated with diphtheria toxoid. They received from 2 to 7 doses, and 21 out of 22 received at least the primary series (3 doses). The last dose of diphtheria toxoid had been administered 20.0 to 38.7 years before.

### Levels of antitetanus toxin IgG (ATT IgG) before vaccination

Geometric mean concentration (GMC) of antitetanus toxin IgG (ATT IgG) in pre-vaccination sera (n = 100) was 832 mIU/ml, ranging from 40 to 15760 mIU/ml. Overall, the proportion immune (≥160 mIU/ml) was 84% and most susceptible women were 70+ years of age (Table 2). GMC in groups A and B were respectively 1291 and 825 mIU/ml but the difference was not statistically significant (p = 0.18).

**Table 2:** Numbers of immune and susceptible women to diphtheria and tetanus, by age group

	Target disease	Age group in years					
			
Immunity (ATT IgG concentration)		30 – 39	40 – 49	50 – 59	60 – 69	70 – 98	Total
	Tetanus						
Immune (≥160 mIU/ml)		16	20	22	12	14	84
Susceptible (<160 mIU/ml)		1	1	3	1	10	16
	Diphtheria						
Immune (≥100 mIU/ml)		12	7	16	11	20	66
Susceptible (<100 mIU/ml)		5	14	9	2	4	34
	Total	17	21	25	13	24	*100

* 100 pre-vaccination sera

**Figure 1:**
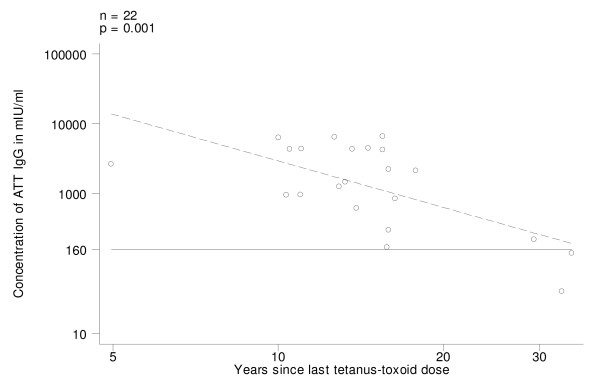
Antitetanus toxin IgG (ATT IgG) levels in pre-vaccination sera, by time since last dose of tetanus-toxoid in women who began vaccination with DTP/PT (Group A). Regression line (stippled) and threshold level of 160 mIU/ml.

**Figure 2:**
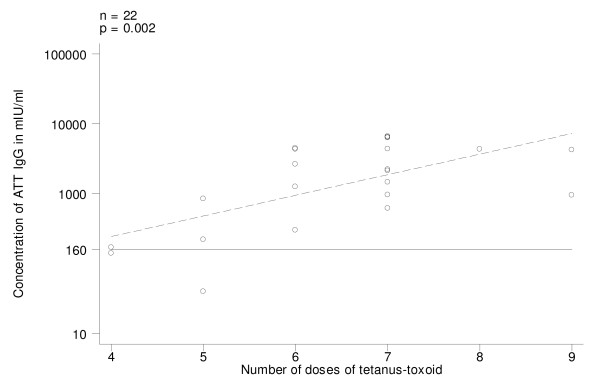
Antitetanus toxin IgG (ATT IgG) levels in pre-vaccination sera, by number of doses of tetanus-toxoid received in women who began vaccination with DTP/PT (Group A). Regression line (stippled) and threshold level of 160 mIU/ml.

In Group A (those who began vaccination with DPT/DT), on univariate analysis, ATT IgG levels decreased with time elapsed since last dose (log transformed variable) (Fig. 1; p = 0.001). All women revaccinated in the last 20 years had ATT IgG above the protective threshold and the 2 susceptible women had been vaccinated more than 32 years before (Fig. 1). ATT IgG levels increased with the number of doses of tetanus toxoid ever received (Fig. 2; p = 0.002). Only 2 women who had received 4–5 doses had ATT IgG below the threshold of 160 mIU/ml (the above mentioned who had received the last tetanus toxoid injection more than 32 years before). On univariate analysis neither present age nor age at first vaccination were associated with ATT IgG levels; age at last vaccination was significant on univariate analysis but was dropped from the final model because it was confounded by the effect of years from last tetanus toxoid. The final model explained 56% of variability of ATT IgG levels and included the total number of tetanus toxoid doses and time since vaccination (Table 3).

**Table 3:** Regression models assessing the association between independent variables and antitetanus toxin levels* in pre-vaccination sera of Group A and Group B women – p values

Independent variable	Group A^a ^(n = 22)		Group B^b ^(n = 66)	
	
	Univariate analysis	Final model	Univariate analysis	Final model
Age in years	0.506	-	<0.001	-
Age at first tetanus toxoid	0.132	-	<0.001	<0.001
N.° doses of tetanus toxoid	0.002	0.022	0.376	-
Years since last tetanus toxoid^c^	0.001	0.016	0.007	0.041
Age at last tetanus toxoid	0.007	-	0.002	-
R^2^	-	0.56	-	0.32

* Log transformation of values in mIU/ml

a: Group A = women who began vaccination with DPT/DT

b: Group B = women who began vaccination with TT

c: This variable was log transformed to be used in the models

**Figure 3:**
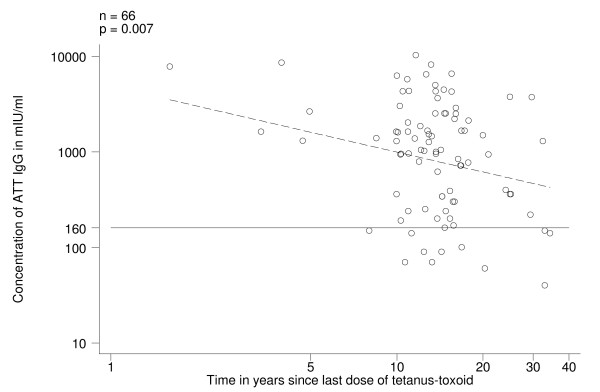
Antitetanus toxin IgG (ATT IgG) levels in pre-vaccination sera, by time since last dose of tetanus-toxoid in women who began vaccination with TT (Group B). Regression line (stippled) and threshold level of 160 mIU/ml.

**Figure 4:**
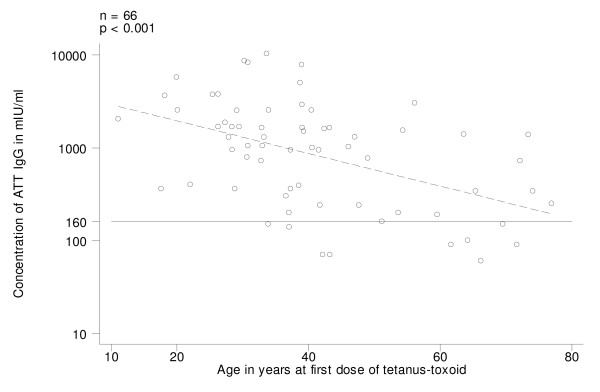
Antitetanus toxin IgG (ATT IgG) levels in pre-vaccination sera, by age at first dose of tetanus-toxoid received in women who began vaccination with TT (Group B). Regression line (stippled) and threshold level of 160 mIU/ml.

In Group B (those who began vaccination with TT), on univariate analysis, ATT IgG levels decreased with time elapsed since last dose (log transformed variable) (Fig. 3; p = 0.007). The levels of ATT IgG were not dependent (p = 0.376) on the number of doses of tetanus toxoid received, but all susceptible women (n = 9) had received less than 4 doses of tetanus toxoid; on the other hand, 8 out of the 9 women who had received 4+ doses of tetanus toxoid (Table 1) had received the last boost more than 10 years before. Furthermore, the decrease of ATT IgG levels with time since last dose was only significant for those who had received 3+ doses. ATT IgG levels were inversely associated with the age at first vaccination (p < 0.001); all 9 women susceptible to tetanus had received the first dose of tetanus toxoid after the age of 33 years (Fig. 4). On univariate analysis, present age and age at last dose were also significantly associated with ATT IgG levels (Table 3) but they were dropped from the final model because of the confounding effect of age at first vaccination. The final model explained 32% of variability of ATT IgG levels and included age at first vaccination with tetanus toxoid and time since the last dose (Table 3).

Pre-vaccination GMC was lower (p = 0.046) among the 12 women without know vaccination history and the proportion of susceptible was higher in this group (p = 0.02). The 5 susceptible women were older than 85 years. Among those 12 women, ATT IgG concentration decreased with age (p = 0.032).

### Levels of antitetanus toxin IgG (ATT IgG) after vaccination

**Figure 5:**
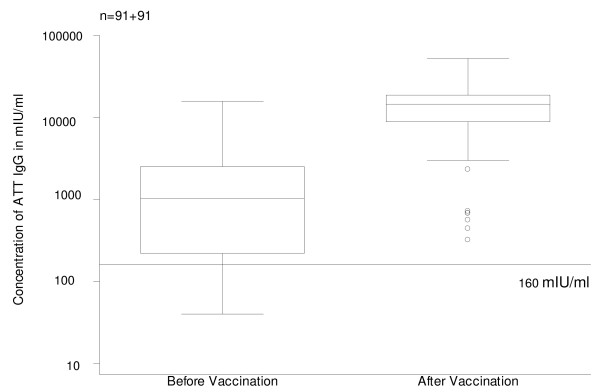
Antitetanus toxin IgG (ATT IgG) levels before and after vaccination. Box plot. Threshold immunity level of 160 mIU/ml.

GMC of ATT IgG among the 91 post-vaccination sera was 11204 mIU/ml, ranging from 320 to 52000 IU/ml. All became immune against tetanus (≥160 mIU/ml; Fig. 5). In only 3 pairs of sera, concentration of ATT IgG was lower among post-vaccination sera; the 3 pre-vaccination levels were extremely high (approximately 1000, 5000 and 150000 mIU/ml). Among the remaining 88 pairs, ATT IgG levels were on average (geometric mean) 9681 mIU/ml higher among post-vaccination sera (compared with pre-vaccination levels), ranging from 200 to 49980 mIU/ml. Post-vaccination ATT IgG levels were highly correlated (p < 0.001) with pre-vaccination levels; when added to the model, age (p = 0.64), time since last dose (p = 0.14) number of previous doses (p = 0.60) and belonging to groups A or B, were not significantly associated with post-vaccination levels.

Following the interpretation proposed by the EIA manufacturer (Table 4), none of the participants needed "basic immunization" and only 37% required "booster vaccination". Following vaccination with Td, most women got "extremely high protection" against tetanus but 2 women still fell in the category "sufficient protection but booster recommended": one of them was 89.7 years old and had no vaccination records; the other was 55.6 years old and had received 3 lifetime doses of tetanus toxoid, with the last one 13.7 years before; this was the only women with lower post-vaccination antibody levels (compared with pre-vaccination levels) both for ATT IgG and ADT IgG.

**Table 4:** Interpretation of IgG antitetanus toxin values observed in 100 pre-vaccination and 91 post-vaccination sera, as proposed by the EIA manufacturer.

Concentration mIU/ml	Vaccination protection	Vaccination recommendation	Before vaccination	After vaccination
<= 30	No	Basic immunization	0	0
]30 – 100]	Not surely guaranteed	Booster vaccination	8	0
]100 – 500]	Existent	Booster vaccination	29	2
]500 – 1000]	Sufficient	Control in 2 years	13	4
]1000 – 5000]	Long term	Control in 5–10 years	41	6
> 5000	Extremely high	Control in 10 years	9	79

### Levels of antidiphtheria toxin IgG (ADT IgG) before vaccination

**Figure 6:**
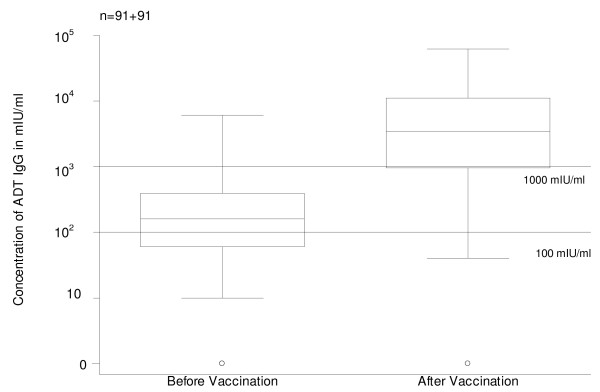
Antidiphtheria toxin IgG (ADT IgG) levels before and after vaccination. Box plot. Thresholds of 100 and 1000 mIU/ml.

In 7 pre-vaccination sera ADT IgG was not measurable (concentration = 0 mIU/ml). Among the remaining 93 sera GMC was 184 mIU/ml, ranging from 10 to 6030. Most women (66%) were immune to diphtheria (ADT IgG ≥100 mIU/ml) though only 6% had long-term protection (ADT IgG ≥1000 mIU/ml) (Table 5 and Fig. 6). The distribution of immunes by age group was clearly different for diphtheria or for tetanus (Table 2). The proportion of women immune to diphtheria varied with age group (p = 0.003; Table 2) with the lower proportion of immunes in the 40–49 years group.

**Table 5:** Interpretation of IgG antidiphtheria toxin values observed in 100 pre-vaccination and 91 post-vaccination sera, as proposed by the EIA manufacturer.

Concentration mIU/ml	Vaccination Recommendation	Before vaccination	After vaccination
< 100	Basic immunisation immediately	34	6
[100 – 1000[	Booster vaccination immediately	60	17
[1000 – 1400[	Booster vaccination after 5 years	3	6
[1400 – 2000[	Booster vaccination after 7 years	1	5
≥ 2000	Booster vaccination after 10 years	2	57

**Figure 7:**
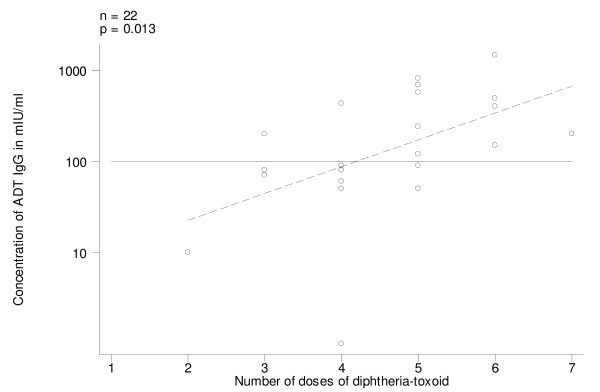
Antidiphtheria toxin IgG (ADT IgG) levels, by number of doses of diphtheria-toxoid administered, in pre-vaccination sera of women who had been vaccinated with diphtheria-toxoid (Group A). Regression line (stippled) and threshold level of 100 mIU/ml.

**Figure 8:**
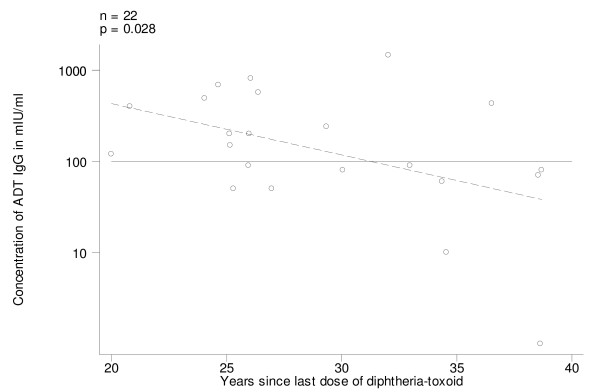
Antidiphtheria toxin IgG (ADT IgG) levels, by time since last dose of diphtheria-toxoid, in pre-vaccination sera of women who had been vaccinated with diphtheria-toxoid (Group A). Regression line (stippled) and threshold level of 100 mIU/ml.

Among the 22 women who had received diphtheria toxoid (Group A), on univariate analysis, ADT IgG levels increased with the number of doses of diphtheria toxoid received (Fig. 7; p = 0.013); all women who had received 6+ doses were immune. On univariate analysis ADT IgG levels were also associated with time since last dose (Fig. 8; p = 0.028) (in the case of diphtheria antibodies there was no need for log transformation of this variable); all susceptible women had received diphtheria toxoid more than 25 years before. It was not possible to assess the relative weight of these two independent variables because they were highly correlated with each other: women receiving fewer doses were the ones that had received the last dose longer ago. Present age was not associated with ADT IgG levels.

Among the 78 women who had never been vaccinated with diphtheria toxoid or which vaccination status was unknown, ADT IgG levels increased with age (p = 0.015). Most of these women (96%) were 40+ years old.

### Levels of antidiphtheria toxin IgG (ADT IgG) after vaccination

In 4 post-vaccination sera ADT IgG was not measurable (concentration = 0 mIU/ml). Among the remaining 87 sera GMC was 2989 mIU/ml, ranging from 40 to 61760. Most women (85/91 = 93.4%) were immune to diphtheria (ADT IgG ≥100 mIU/ml) though only 74.7% (68/91) had long-term protection (ADT IgG ≥1000 mIU/ml) (Table 5 and Fig. 6). In 7 pairs of sera, concentration of ATT IgG was the same or lower among post-vaccination sera. Among the remaining 84 pairs, ADT IgG levels were on average (geometric mean) 2983 mIU/ml higher among post-vaccination sera (compared with pre-vaccination levels), ranging from 20 to 61570 mIU/ml. Post-vaccination ADT IgG levels were highly correlated (p < 0.001) with pre-vaccination levels; when added to the model, age was not significantly (p = 0.24) associated with post-vaccination levels.

Following the interpretation of the ELISA manufacturer (Table 5), the 6 women with levels below 100 mIU/ml after vaccination with Td, should receive "basic immunization immediately". All had just received one dose of diphtheria toxoid in their lives.

## Discussion

Vaccination history was highly dependent upon age, which had already been found in countries with old national vaccination programmes [4]. Groups named A and B correspond to two generations of Portuguese citizens. One group is younger and was "covered" in their childhood by the existence of the NVP, while in the other group (B) most women were more than 10 years when the programme began. Though the differences in antibody concentrations (for both pre and post-vaccination ATT IgG and ADT IgG), between these two groups, were not statistically significant, the factors that affected the ATT IgG levels in pre-vaccination sera were different.

For those who began vaccination in childhood with DPT/DT (Group A), the total number of doses and time since last vaccination were determinant factors; all had received a complete primary series (3 doses) and at least one booster and no susceptibles were observed before 20 years had elapsed from the last dose. Our findings are consistent with those from Simonsen [4].

Among Group B most women began vaccination in adulthood, with TT, and ATT IgG decreased with time since vaccination and age at first dose, which partially agrees with the mentioned Danish study [4]. In this group, several women who had received their last dose more than 10 years before were susceptible and all women who had begun vaccination before 30 years of age were immune. The difference between Groups A and B in what concerns the effect of time elapsed from the last dose, maybe due to similar kinetics but longer duration of immunity among those who receive boosters, relative to those who only received the primary series (or less than that), already observed by other authors [17]. We were surprised to find no association with the number doses received within this group, but that may be explained by the fact that most women had not even completed the 3 doses plus 2 boosters, recommended by the Portuguese NVP guidelines [20]. Furthermore, the decline in protective antibody levels with time since vaccination was only significant among those who had received 3+ doses, which again is in favour of previous findings of insufficient protective effect of less than 3 doses [1,17].

The distribution of protective antibody levels against diphtheria also seems to show the existence of two different population groups. Among women vaccinated in their childhood (Group A), most were younger less than 40 years of age (14/22); variation in ADT IgG pre-vaccination levels was associated with the number of doses and time elapsed from the last dose, showing antibody kinetics similar to that of ATT IgG, which had already been mentioned in the literature [4]. Nevertheless it was surprising that only those vaccinated more than 30 years before were susceptible. Like it has been hypothesized to explain similar observations in Canada [4,13], naturally acquired immunity may have contributed to maintain immunity after vaccination, in some of the vaccinated women we have studied. In the other group (Group B plus the 12 women without vaccination history) most women were 40+ years old (75/78) and ADT IgG levels increased with age, consistent with the findings from the Portuguese national serological survey (NSS) performed in 2001/2002 [24]. These women belong to a generation who lived in a time when diphtheria incidence was high in Portugal and they may have been naturally immunised.

Response to vaccination (or revaccination), measured as concentration in post-vaccination sera or as difference in concentration between post and pre-vaccination sera, depended on the pre-vaccination concentration levels, both for tetanus and diphtheria, which is again in agreement with previous published findings [4]. We did not find significant influence of present age or age at first vaccination on the post-vaccination antibody response, which is not in agreement with previous findings [17]. We cannot find a reason for that negative finding besides the wide variation of pre-vaccination levels, which in its turn could be due to factors not assessed in this study. Nevertheless our study confirms that, even in old people, safe protective ATT IgG levels can be attained by revaccination [1,17,29].

The post-vaccination ADT IgG levels lower in what concerns protection, relative to tetanus, might be due to the lower pre-vaccination levels of ADT IgG. On the other hand, for almost all women vaccination with Td was a booster for tetanus protection, while it was the first dose of diphtheria toxoid ever received; even for those few women in which diphtheria toxoid was a booster, longer periods of time had elapsed from last vaccination.

## Conclusion

Added to previous evidence published in the literature elsewhere, our study suggests that, to protect against tetanus, there is no need to administer decennial boosters to the Portuguese adults who have complied with the aggressive childhood/adolescent schedule, which includes 6 doses of tetanus toxoid. The adult booster intervals could be wider, probably of 20 years. This also seems to apply to protection against diphtheria, but issues on the herd immunity effect and on the circulation of toxigenic strains need to be better understood.

On the other hand, our study shows that many Portuguese adult women born before the beginning of the NVP are not adequately vaccinated against tetanus. That is particularly evident among women 70+ years of age. The analysis of the few sporadic cases of tetanus, especially among elderly women, reported in Portugal in the beginning of this century, had shown that missed oportunities to vaccinate are a problem [30]. In this generation, though the duration of immunity following the primary series also seems to be longer than ten years, the priority is completing the primary series and first boosters as recommended.

## Competing interests

The author(s) declare that they have no competing interests.

## Authors' contributions

GG contributed to study design, data analysis and interpretation, entered data in a computer database, and leaded the manuscript development. MAS contributed to study design, carried out the immunoassays, and participated in the manuscript development. JGF contributed to study design, enrolled and vaccinated the participant women, collected data on vaccination status, entered data in a computer database, and critically revised the manuscript. JSC contributed to study design, supervised data collection procedures, and participated in manuscript development. All authors read and approved the final manuscript.

## Pre-publication history

The pre-publication history for this paper can be accessed here:


